# Comparison of Serological and Molecular Methods for Detection of *Spiroplasma citri* in Moroccan Citrus-Growing Areas

**DOI:** 10.3390/plants12030667

**Published:** 2023-02-02

**Authors:** Tourya Sagouti, Naima Rhallabi, Giancarlo Polizzi, Abdessalem Tahiri, Zineb Belabess, Essaid Ait Barka, Rachid Lahlali

**Affiliations:** 1 Laboratoire de Virologie, Microbiologie et Qualité/Ecotoxicologie et Biodiversité, Faculté des Sciences et Techniques de Mohammedia, Mohammedia 20650, Morocco; 2 Dipartimento di Agricoltura, Alimentazione e Ambiente, sez. Patologia Vegetale, University of Catania, Via S. Sofia 100, 95123 Catania, Italy; 3 Phytopathology Unit, Department of Plant Protection, Ecole Nationale d’Agriculture de Meknès, Km 10, Rte Haj Kaddour, BP S/40, Meknes 50001, Morocco; 4 Plant Protection Laboratory, Regional Center of Agricultural Research of Meknes, National Institute of Agricultural Research, Km 13, Route Haj Kaddour, BP. 578, Meknes 50000, Morocco; 5 Unité de Recherche Résistance Induite et Bio-Protection des Plantes-EA 4707, Université de Reims Champagne-Ardenne, 51100 Reims, France

**Keywords:** citrus stubborn disease, *Spiroplasma citri*, Morocco, DAS-ELISA, PCR, real-time PCR

## Abstract

*Spiroplasma citri*, a helical motile, wall-less, and cultivable microorganism of the class Mollicutes, is the agent of the citrus stubborn disease. There is currently a lack of data about the presence of this pathogen in Moroccan citrus orchards. This study aims to validate serological and molecular methods for routine *S. citri* diagnosis in Moroccan citrus groves. To provide an update on the present status of the outbreak of the pathogen in Moroccan citrus orchards, a survey of *S. citri* was conducted in the main citrus-growing regions of Morocco. A total of 575 leaf samples were collected from citrus trees with symptoms attributable to *S. citri* infection. Samples were collected during 2020 and 2021 from 23 citrus orchards. The presence of *S. citri* was tested in all samples using the double antibody sandwich enzyme-linked immunosorbent assay (DAS-ELISA). Using this method, 57 samples were found to be infected with *S. citri*, 41 samples had doubtful results, and the remaining samples were negative. To corroborate the results of the DAS-ELISA test, 148 samples were chosen for additional molecular testing using conventional polymerase chain reaction (PCR) and real-time PCR (qPCR) based on specific primer pairs targeting three different genes (putative adhesion-like gene P58, putative adhesion gene P89, and spiralin gene). Using primers that target the putative adhesion-like gene P58, *S. citri* was detected by conventional and real-time PCR amplification from plant tissue with differing degrees of specificity. The results allowed us to determine the incidence of *S. citri* in all Moroccan citrus orchards, with a wide range of positive samples varying from 6.5% to 78%, and to show that molecular tests, particularly real-time PCR assays that target the putative adhesion-like gene P58, are the most sensitive for making an accurate diagnosis of *S. citri*. Indeed, the real-time PCR with P58-targeting primers yielded positive results from all positive and doubtful ELISA samples as well as some negative samples, with an OD value close to 1.5× times healthy samples, thus demonstrating a high sensibility of this technique.

## 1. Introduction

Citrus is one of the most significant fruit crops grown nowadays in Morocco. The national citrus production area comprises 126.600 ha, and 2.6 million tons of various citrus fruits are produced each year [1]. However, citrus stubborn disease (CSD) continues to pose a severe danger to the citrus fruit industry and damage citrus production [2]. CSD is a significant disease of young citrus plants in numerous hot and arid citrus growing zones [3]. The causal agent of CSD *is Spiroplasma citri*, a helical motile, wall-less, and cultivable microorganism that belongs to the class Mollicutes [3]. The pathogen is phloem limited, leafhopper transmitted [4,5], and also graft transmissible [6,7,8]. In addition to citrus species, the mollicute infects a wide range of non-rutaceous plant species such as carrot (*Daucus carota subsp. sativus*) [9], periwinkle plants (*Catharanthus roseus*) [10], and China aster (*Callistephus chinensis*) [11].

The disease was reported in several areas and countries, including California, Arizona, North Africa, the eastern Mediterranean Basin, and the Middle East [5]. Although the disease is rarely fatal, affected trees can be severely stunted, showing dense and unusually upright leaves. In Riverside, California, reports of CSD’s fluctuating citrus production losses in CSD affected trees ranging from 44 to 74% for Valencia oranges and up to 100% for Navel oranges [12]. In addition, CSD affects fruit production and yield in several ways. Navel orange trees infected with *S. citri* produced 26 to 32% fewer fruits than did *S. citri*-free trees [13].

**Figure 1:**
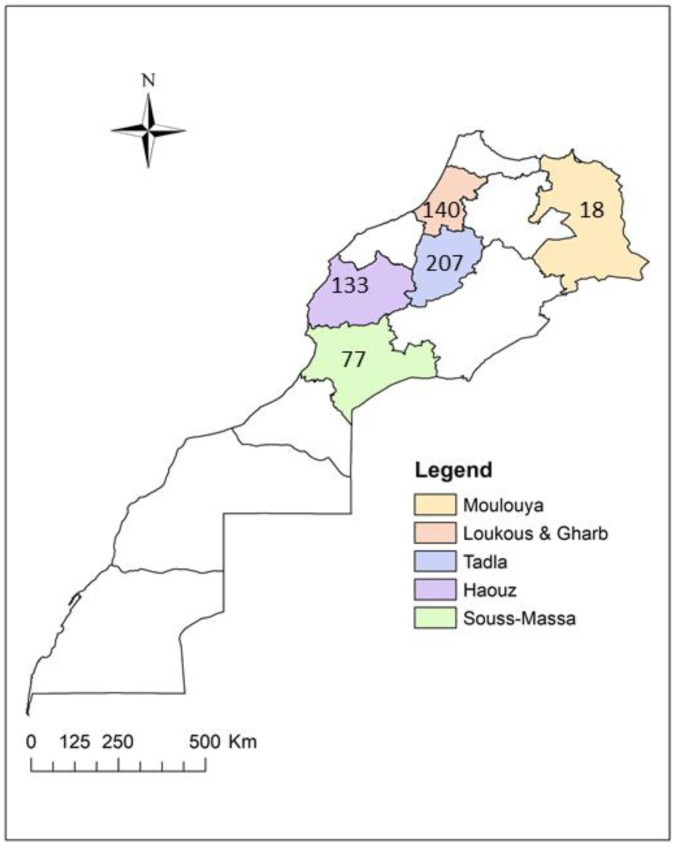
Map showing the six Moroccan regions (indicated by colored areas) where field surveys were carried out to monitor the spread of *Spiroplasma citri*. The numbers in the colored regions show how many samples were taken in each area.

In Morocco, Wyss-Dumont [14] and Perret [15] reported CSD for the first time in 1951, and more studies later confirmed the disease occurrence in the country [16]. In all Moroccan citrus-growing regions, including Gharb, Haouz, Loukkos, Moulouya, Tadla, and Souss (Figure 1), *S. citri* has been detected [17,18]. In 1970, the disease was noted in the Tadla and Souss regions. There were so many severely damaged trees in the Tadla region that entire orchards had to be cut down. More recently, Afechtal [19] confirmed the presence of CSD in Moroccan citrus orchards. However, to our knowledge, to date, no other research has reported the distribution of CSD in the different citrus-producing regions of Morocco.

Enzyme-linked immunosorbent assay (ELISA) is a diagnostic method frequently used to detect *S. citri* in infected material [4,20]. However, additional DNA-based techniques have been developed, such as conventional polymerase chain reaction (PCR) and real-time PCR (qPCR), which use primers that target the spiralin and adhesion genes [3,21]. These methods were employed for the detection of *S. citri* in plant tissues and liquid cultures of *S. citri*. Utilizing sequence analysis of nucleotide fragments and comparison with various isolates from around the world, *S. citri* detection was greatly improved [22,23]. Numerous sensitive and reliable qPCR tests were developed to avoid the lack of sensitivity of *S. citri* detection caused by the erratic distribution and low titers of *S. citri* DNA in diseased trees [3,24].

A technical regulation governing the control of certified plants requires the performance of serological and molecular analysis to confirm that citrus plants are free of viruses, viroids, and mycoplasmas, including *S. citri* [25]. Several techniques and methods are currently used for the identification of these diseases, such as serology, which is the main technique used, followed by biological tests [26], while molecular tests are not well developed, and hence the importance of comparing the efficiency of molecular and serological tests to strengthen the certification system of Moroccan plants.

The objectives of this study were (i) to determine the prevalence of CSD in citrus orchards and provide an updated picture of its spread in Morocco, and (ii) to improve the phytosanitary control system for certified plants and the monitoring of the phytosanitary situation of Moroccan citrus orchards by comparing the efficiency of double antibody sandwich ELISA (DAS-ELISA) tests with molecular approaches employing both PCR and qPCR.

## 2. Results

### 2.1. Symptoms of CSD

**Figure 2:**
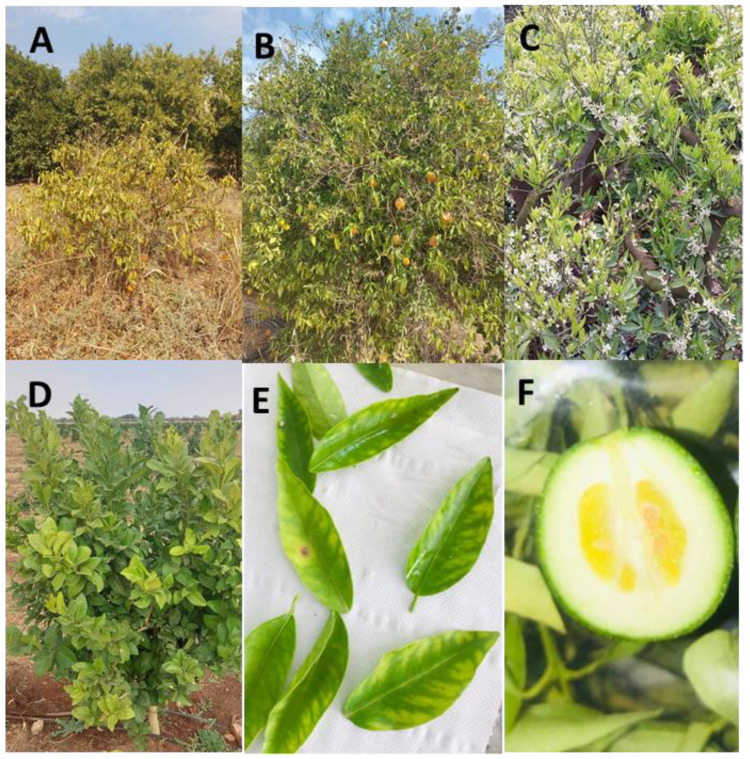
Field symptoms of citrus stubborn disease observed in sweet orange trees (*Citrus sinensis*) in the Tadla region in the growing season of 2021. (**A**,**B**) Stunted Navel sweet orange trees, (**C**) tree with off-season flowering, (**D**) infected young plant of sweet orange on sour orange rootstock, (**E**) leaf chlorosis, (**F**) Navel sweet orange fruit showing a gland shape.

The majority of the prospected orchards displayed CSD symptoms, indicating that the disease is common throughout Moroccan citrus-growing regions, particularly in the Tadla and Gharb. Infected citrus trees showed branch mottling, yellowing, the proliferation of many buds, chlorosis of the leaves, and the production of tiny and irregularly shaped fruits (lopsided or corn-shaped) (Figure 2).

### 2.2. Detection of Spiroplasma citri by DAS-ELISA

To discriminate between positive, negative, and doubtful samples, a preliminary screening using DAS-ELISA was performed. *S. citri* was found in plant tissues from citrus field trees with this method. Of the 575 leaf samples tested, approximately 10% (57) produced positive ELISA results. However, 7% (40/575) of the analyzed samples produced doubtful results. The remaining 478 samples (83%) were all negative. Positive samples were found in the six citrus-growing areas that were surveyed (Table 1). *S. citri* was most prevalent in orange (*Citrus sinensis* (L.) Osbeck) trees, followed by clementine (*Citrus clementina* hort. ex Tanaka), lemon (*Citrus limon* (L.) Burm. f), and mandarin (*Citrus reticulata* Blanco) (data not shown).

### 2.3. Detection of Spiroplasma citri by PCR and qPCR

The results of the serological and molecular analysis of the tested samples are presented in Table 1. Validation of the DAS-ELISA result was conducted using the molecular PCR method and qPCR. Positive (57), doubtful (40), and approximately 10% (50/478) of the negative DAS-ELISA samples were retested with both the conventional PCR and qPCR methods using different primer pairs.

**Figure 3:**
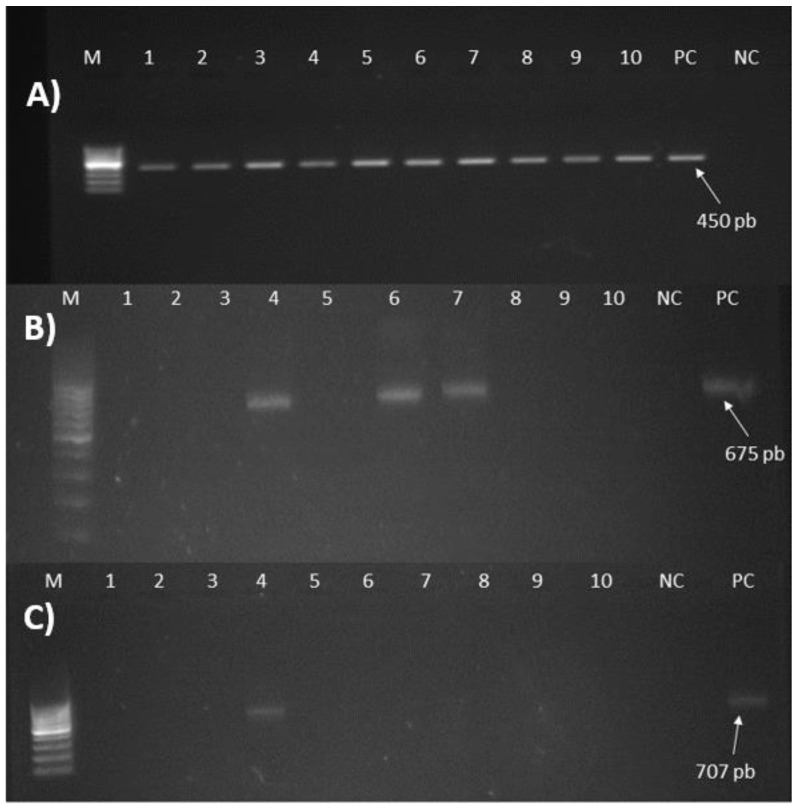
Amplification products of PCR tests targeting three different genes of *Spiroplasma citri*. Lane NC is a negative control; lane PC is a positive control; and lane M corresponds to the DNA ladder (Invitrogen, Waltham, MA, USA). (**A**) Products generated with the primer pair targeting the putative adhesion gene P58. Lanes 1–10: 10 samples tested positive. (**B**) Products generated with the primer pair targeting the spiralin gene. Lanes 3, 5, and 6: samples tested positive. Lanes 1–2, 4, and 7–8: samples tested negative. (**C**) Products generated with the primer pair targeting the putative adhesion gene P89. Lane 4: the only sample tested positive. Lanes 1–3 and 5–10: samples tested negative.

PCR using primers targeting the putative adhesin gene P58 produced amplicons of the expected size (450 bp) (Figure 3) from all samples that have already reacted positively with the DAS-ELISA test. Moreover, this test allowed us to obtain positive samples among those which were doubtful and negative with the DAS-ELISA. Indeed, positive results were obtained for 61% and 16% of the DAS-ELSIA-doubtful and negative samples, respectively.

PCR using primers targeting the spiralin gene produced amplicons with the expected size (675 bp) from only 5% of samples (3/57) that previously showed positive results with the DAS-ELISA assay (Figure 3). Similarly, only a small number of samples tested positive with this test from samples that were considered doubtful (5%, 2/41) and negative (6%, 3/50) with the DAS-ELISA test.

Conventional PCR using primers targeting the putative P89 adhesin gene produced an amplicon of the expected size (707 bp) for only 2% (1/57) of samples that had previously been detected as positive with the DAS-ELISA test (Figure 3).

The qPCR using putative P58 adhesin gene-specific primers showed the same finding for positive samples with DAS-ELISA. Similarly, the results of the qPCR tests indicated that 93% of doubtful samples in the DAS-ELISA category were positive. Surprisingly, 28% (14/50) of samples that were negative for DAS-ELISA tested positive with this technique (Table 1).

The results highlighted that only 37% (21/57) of the DAS-ELISA-positive samples yielded positive results in the qPCR test targeting the spiralin gene. In addition, 24% (10/41) of doubtful samples and 2% (1/50) of negative samples with DAS-ELISA were detected as positive with this technique (Table 1).

**Figure 4:**
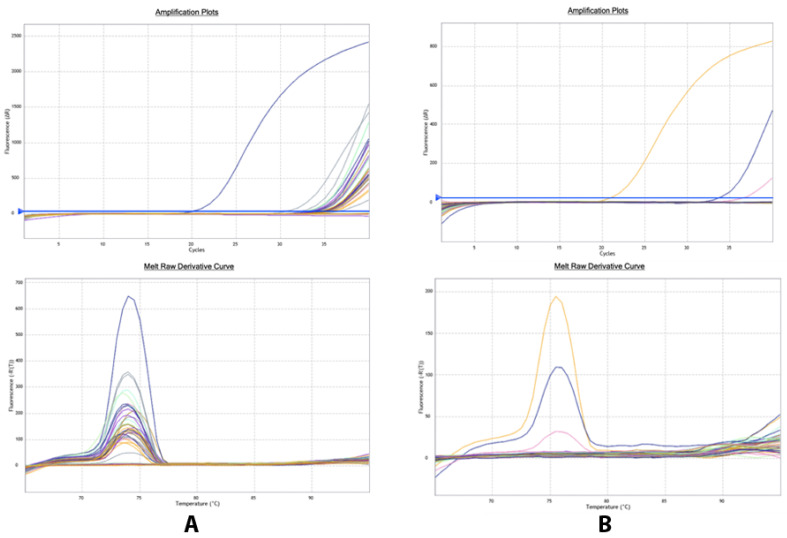
qPCR for *Spiroplasma citri* detection. Amplification plot and melt peak analysis showing test result using (**A**) P58 primers and (**B**) spiralin primers, the colors refers to the samples.

Compared to all used tests, the qPCR using P58 primers was shown to be the most sensitive in making an accurate diagnosis of *S. citri*. Indeed, this test could detect all positive and doubtful samples from ELISA as well as a portion of negative samples with an OD value near 1.5 times healthy samples, indicating a high sensibility of this technique. A sample is considered positive if the cycle threshold (Ct) is less than 32 (Table 2). Additionally, it has been shown through a comparison of the qPCR detection methods that the P58 gene-targeting primer pair detects more positive samples than the spiralin primers (Figure 4).

*S. citri* was most commonly detected in the Moulouya region where 83% of the tested samples were positive. This was followed by Loukkos, where more than half of the tested samples were positive (57%), and Tadla, where 21% of the tested samples were positive, as well as Haouz, Gharb, and Souss (Table 1).

All positive ELISA samples were confirmed by a qPCR test targeting the P58 gene; however, 83% and only 27% of doubtful and negative ELISA samples were qPCR positive, respectively. The average Ct from every category is demonstrated in Table 2.

### 2.4. Nucleotide Sequencing Analysis

The results of BLAST analysis confirmed that the obtained sequence of the isolate (BERK1) was identical to *S. citri* based on the putative adhesin P58 gene. The nucleotide sequence was deposited into GenBank with accession number “OM304995” as a *Spiroplasma citri* BERK1 isolate. The BERK1 isolate was closely related to the Syrian isolate strain Y12 (Acc. No. FN555141) via 99.64% and the USA isolate (Acc. No. EU602315) via 98.99%.

## 3. Discussion

The presence of *S. citri* in Moroccan orchards was previously reported in several studies [17,19,27,28]. However, no study has focused on the incidence of this pathogen in Moroccan citrus growing areas and diagnostic methods for its detection.

The current study is extremely relevant for the citrus industry since it provides a current picture of the status of CSD in Moroccan citrus orchards, in contrast to previous studies that only reported the detection of *S. citri* in Morocco [19]. Compared to previous research, this investigation reports the analysis results performed on a large number of samples from the six citrus-growing regions of Morocco. In addition, in this study, the comparison of the sensitivity of the serological and molecular detection techniques, including conventional PCR, qPCR, and DAS-ELISA, was made. The samples were first analyzed using the DAS-ELISA, and those found to be positive or doubtful and a portion of 10% negative samples were subsequently put through additional molecular testing, including conventional PCR and qPCR. Indeed, we report the prevalence of *S. citri* in the principal citrus-growing regions of the country. It is crucial to note that this study is the first to use these three detection techniques to detect and describe the distribution and incidence of *S. citri* in the major Moroccan citrus-growing areas.

Serological methods have been employed for several years to regularly detect *S. citri* [20,29], particularly within the framework of regulatory phytosanitary control of certified Moroccan citrus plants [25,26]. Using DAS-ELISA, it was shown that *S. citri* could be detected in infected host plant tissue, arthropod hosts, and pure culture [30,31,32]. Due to its simplicity of use and capacity to assess a large number of samples, ELISA has been the most often employed diagnosis method in the preliminary sanitary evaluation of propagating material [33]. Therefore, we used DAS-ELISA to monitor the incidence of *S. citri* in Moroccan citrus orchards. Our findings show that the DAS-ELISA test is a useful technique for screening a large number of samples, classifying them, and testing a smaller number of samples by PCR for confirmation.

Our findings show that the putative P58-adhesin-gene-targeting set of primers provides the greatest *S. citri* detection results in both conventional and real-time PCR. PCR using primers targeting the putative P58 adhesin gene produced amplicons to the expected size from all samples that yielded positive results with the DAS-ELISA test. These primers were also able to identify positive samples among those yielding doubtful and negative results with DAS-ELISA and to confirm the infection status of the positive samples with the same test. Regarding DAS-ELISA negative and doubtful samples, it is significant to notice that qPCR recovered more positive samples than conventional PCR. This time, the putative P58-adhesin-gene-specific qPCR primers raised the same finding. Every sample that responded positively to the DAS-ELISA test did so when the qPCR test was performed on it. The qPCR test similarly indicated that 93% of the samples in the DAS-ELISA category with doubtful results were infected. Additionally, this test allowed for the identification of positive samples among those falling into the DAS-ELISA group of negative samples; 28% of the samples tested yielded positive results. This might mostly be explained by the fact that ELISA is known to have a detection sensitivity lower than DNA-based methods [18,30,34] due to the low titers of the pathogen or because of its uneven distribution in the plant and low titers in diseased trees, as well as considerable seasonal fluctuations [7]. The increased sensitivity of qPCR has additional value for the early detection of the disease agent [21,24,35].

The primer pair targeting the P58 gene showed better sensitivity for the detection of Moroccan *S. citri* strains than primers targeting the P89 and spiralin genes. This resulted in a *S. citri* incidence varying from 6.5% (Souss) to 78% (Moulouya). This variability in primer efficiency among *S. citri* isolates has been reported previously in Egypt [23,36] and Turkey [22]. This result has already been reported previously [3,22,36]. In other words, it was already known that spiralin-targeting primers were less sensitive for *S. citri* detection. This could be a result of the uneven distribution and low titers of *S. citri* found in field samples, as well as the single copy of the spiralin gene found in the genome of all *S. citri* and its single copy on each chromosome [35,37,38]. P58, a putative adhesin multigene of *S. citri*, on the other hand, most likely has numerous copies present.

In the present study, *S. citri* was detected in samples collected from orange and clementine trees. This result is consistent with subsequent studies revealing that orange Navel (*C. sinensis*) is the cultivar that is most susceptible to *S. citri* infection [39,40,41].

## 4. Materials and Methods

### 4.1. Plant Samples and Tissue Preparation

Leaf samples were collected from the upper canopy of the tree in the four cardinal directions and the middle. Leaf samples from the same tree were pooled to generate one leaf sample for analysis. In total, 575 leaf samples were collected from 575 citrus trees between July 2020 and October 2021 from the six main citrus-growing areas in Morocco: 77 from Souss, 140 from Loukkos and Gharb, 18 from Moulouya, 133 from Haouz, and 207 from Tadla (Figure 1). Prelevement was essentially oriented at symptomatic trees that exhibited conspicuous CSD symptoms (Figure 2). Leaf samples were generally collected from different citrus species, including orange (*C. sinensis*), clementine (*C. clementina*), mandarin (*C. reticulata*), and lemon (*C. limon*).

For experimental analysis, petioles plus the main vein of leaf samples (five grams) were placed in a mesh bag containing 4 mL of 1 × commercial extraction buffer (PBS) provided by the supplier (Agdia EMEA, Soisy sur seine, FRANCE). The Homogenizer HOMEX (Bioreba AG, Reinach, Swiss) instrument was used for crushing plant material. Each sample’s ground material was collected in a 1.5 mL tube and centrifuged at 5000 rpm for 5 min. The resulting supernatant was used for serological and molecular detection.

### 4.2. DAS-ELISA

For the DAS-ELISA analysis assay, 100 µL of the supernatant from the plant juice sample was used. The test was carried out according to the manufacturer’s instruction (Agdia EMEA, Soisy sur seine, FRANCE), which is based on the use of polyclonal antibody alkaline phosphatase conjugate. Each test plate had two replicates of each sample as well as two wells for positive and negative controls (buffer only and healthy control sample). Absorbance was measured after 1 and 2 h of incubation with the p-nitrophenyl phosphate substrate at 37 °C using a BioTek 800 TS absorbance reader that detects optical density (OD) at 405 nm. Samples were determined to be positive if the absorbance, i.e., OD was two times greater than the mean absorbance of the threshold calculated based on the OD of healthy control (HC). A sample is positive if its OD > 2 times the threshold calculated based on the OD of HC. However, a sample is considered doubtful if its OD was between 1.5 to 2 times greater than the OD of HC.

### 4.3. Genomic DNA Extraction

A total of 148 samples (positive, doubtful, and a portion of negative samples with high OD from the DAS-ELISA test) were selected for genomic DNA extraction. The genomic DNA was directly extracted from 200 µL of the same supernatant used previously in the ELISA test. DNA extraction was performed with the MagListoTM 5M Genomic DNA Extraction Kit (Bioneer, Daejeon, Korea) following its specific protocol. The DNA was then stored at −20 °C until use.

### 4.4. PCR

For conventional PCR assay analysis, three specific primer pairs that target the spiralin gene [35], putative adhesion gene P58, and putative adhesion gene P89 [3] were used to detect *S. citri* in the selected samples.

The PCR reaction mixture was performed in a total volume of 25 µL, which contained 2 µL of template DNA, 0.5 µM of each primer (10 µM), 12.5 µL of a HotGoldStar Mix (Eurogentec, Liege, Belgium), and 7.5 µL of deionized PCR-grade water. The amplification consisted of a denaturation step at 95 °C for 10 min followed by 40 cycles of 30 s at 95 °C, 30 s at 56 °C, 90 s at 72 °C, and a final extension step of 5 min at 72 °C [3]. PCR products were then checked on a 1.5% agarose gel by electrophoresis using TAE buffer (×1), stained using Sybersafe, and visualized using a UV trans-illuminator. Samples were considered positive if a band of the expected size was visualized.

### 4.5. qPCR

The qPCR assay was performed in an AriaMx Real-Time PCR System (Agilent, CA, USA) using SyberGreen qPCR Mix (Applied Biosystems, CA, USA). Two specific primer pairs targeting the spiralin gene [42] and putative adhesion gene P58 [21] were used. Each reaction mixture contained 2 µL of genomic DNA, 0.8 µM of each primer, and 10 µL of 2 × Power SyberGreen qPCR Mix and deionized PCR-grade water for a final volume of 25 μL. The thermal profile consisted of one step at 95 °C for 10 min, followed by 35 cycles of 95 °C for 15 s, and 61 °C for 60 s. Melting curve: 60 °C to 95 °C at melt rates of 0.5 °C/10 s [21]. A sample is considered positive if it produces an exponential curve amplification with threshold values < 32 and has a melt curve showing the same peak as the positive control [3].

### 4.6. Nucleotide Sequencing Analysis

To confirm the identification of *S. citri,* one PCR-positive sample representing the Moulouya region was subjected to further molecular characterization by sequence analysis of the putative adhesion-like gene P58. The sample was sequenced by “STAB VIDA Company” using Sanger sequencing. The sequence was edited and aligned using DNAMAN sequence analysis software (version 6.0, Lynnon Biosoft, CA, USA), verified using Blast search to identify similar sequences in GenBank databases (National Center for Biotechnology Information), and submitted to the Gen Bank.

## 5. Conclusions

This study emphasizes the significance of complementary PCR and qPCR analysis along with serological testing for the detection of *S. citri* in the surveillance program of Moroccan citrus orchards to manage the CSD and prevent citrus production losses. In conclusion, even though the pathogen titer was low in the samples collected, the results of this analysis will improve citrus certification programs and the management of CSD through early diagnosis. They will also shed light on the true spread and distribution of CSD in Moroccan orchards. In-depth research might be conducted on the additional biological and molecular characterization of *S. citri* isolates representative of the various citrus-growing regions. Additionally, to increase the sensitivity of early detection techniques, particularly in the context of the control of certified plants, it is critical to develop molecular diagnostic tools specifically for Moroccan isolates.

## Figures and Tables

**Table 1:** DAS-ELISA and PCR test results of leaf samples collected across several citrus-growing areas in Morocco from July 2020 to October 2021.

Region		Serological				Adhesine P58 Gene		Spiralin Gene	
	Analyzed Samples	Negative	Suspect	Positive	Analyzed Samples *	PCR	qPCR	PCR	qPCR
TADLA	207	168 (81.16%)	14 (6.76%)	25 (12.08%)	61	36 (59.02)	43 (70.49%)	2 (4.65%)	13 (21.31%)
GHARB	126	111(88.10%)	5 (3.97%)	10 (7.94%)	19	14 (73.68)	15 (78.95%)	2 (13.33%)	3 (15.79%)
HAOUZ	133	115 (86.47%)	12 (9.02%)	6 (4.51%)	33	14 (42.42)	20 (60.61%)	1 (5%)	6 (18.18%)
SOUSS	77	73 (94.81%)	2 (2.60)	2 (2.60%)	7	3 (42.86)	5 (71.42%)	0 (0%)	1 (14.29%)
LOUKKOS	14	7 (5%)	3(21.43%)	4 (28.57%)	10	7 (70%)	8 (80%)	0 (0%)	6 (60%)
MOULOUYA	18	4 (22.22%)	7 (38.88%)	7 (38.88%)	18	13 (72.22%)	15 (83.33)	1 (6.67%)	3 (6.67%)
Total	575	478 (83.13%)	43 (7.48%)	54 (9.39%)	148	87 (58.78%)	106 (71.62%)	6 (4.05%)	32 (21.62%)

* Positive, doubtful, and a portion of negative samples with high OD from the DAS-ELISA test.

**Table 2:** Average Ct of qPCR positives by category of ELISA-positive samples.

	Number of Positive Samples					
Region	Positive-ELISA	qPCR (P58)/Average Ct	Doubtful ELISA Samples	Positive qPCR (P58)/Average Ct	Negative ELISA Samples	Positive qPCR (P58)/Average Ct
SOUSS	3	3 (average Ct = 29.2)	2	2 (average Ct = 30.5)	2	0
TADLA	25	25 (average Ct = 28.6)	14	12 (average Ct = 30.5)	22	6 (average Ct = 31.4)
LOUKKOS	4	4 (average Ct = 29.2)	3	3 (average Ct = 30.3)	3	1 (average Ct = 32)
MOULOUYA	7	7 (average Ct = 29.8)	7	4 (average Ct = 30.5)	4	2 (average Ct = 31.8)
GHARB	10	10 (average Ct = 28.76)	5	4 (average Ct = 29.8)	4	1 (average Ct = 31)
HAOUZ	5	5 (average Ct = 29.2)	12	11 (average Ct = 30.7)	16	4 (average Ct = 31.8)
Total	54	54/54	43	36/43	51	14/51

## Data Availability

Not applicable.
